# GSK-3 and mitochondria in cancer cells

**DOI:** 10.3389/fonc.2013.00016

**Published:** 2013-02-05

**Authors:** Federica Chiara, Andrea Rasola

**Affiliations:** ^1^Department of Molecular Medicine, University of PadovaPadova, Italy; ^2^Institute of Neuroscience, Consiglio Nazionale delle Ricerche, University of PadovaPadova, Italy; ^3^Department of Biomedical Sciences, University of PadovaPadova, Italy

**Keywords:** GSK-3, mitochondria, ROS, chemotherapeutics, PTP, cancer, apoptosis

## Abstract

GSK-3 is a multifunctional kinase that is located in the cytosol, nucleus, and mitochondria of all cell types, and it is involved in the pathogenesis of a variety of diseases. In cancer, GSK-3 modulates the response of the cell death machinery to stress stimuli, including chemotherapeutics. Mitochondria are at the heart of the integration between survival and noxious signals; therefore, modulation of the mitochondrial functions carried out by GSK-3 is profoundly involved in the apoptosis escape capabilities that hallmark neoplasms. This review briefly covers the mechanistic interactions among oncogenic kinase pathways, GSK-3 activity and subsequent modulation of mitochondrial functions that shape the pro-survival phenotype of cancer cells, such as control of redox homeostasis and inhibition of the mitochondrial permeability transition pore.

## INTRODUCTION

GSK-3 is a Ser/Thr protein kinase ubiquitously expressed and extremely conserved in all cell types of every eukaryotic species examined to date. There are two GSK-3 isoforms, GSK-3α and GSK-3β, which are highly homologous and have been implicated in a variety of critical regulatory roles. Little is known about the respective functional roles of the two kinase isoforms, and GSK-3α and GSK-3β are largely interchangeable (Juhaszova et al., 2009). Thus, for the sake of simplicity, we will discuss about GSK-3 functions without discriminating between the two isoenzymes, even if most studies of GSK-3 and mitochondria refer to GSK-3β.

At variance from the majority of kinases, GSK-3 is constitutively active, and more than 50 substrates have been identified. Dysregulation of GSK-3 is linked to a large number of prevalent diseases including psychiatric disorders, neurodegenerative diseases, ischemia/reperfusion injury, diabetes, and cancer (Jope and Johnson, 2004; Miura et al., 2010; Medina et al., 2011). In the present review we will analyze the role played by GSK-3 in tumor cells, with a particular focus on biological processes involving mitochondria.

## GSK-3 REGULATION

**FIGURE 1:**
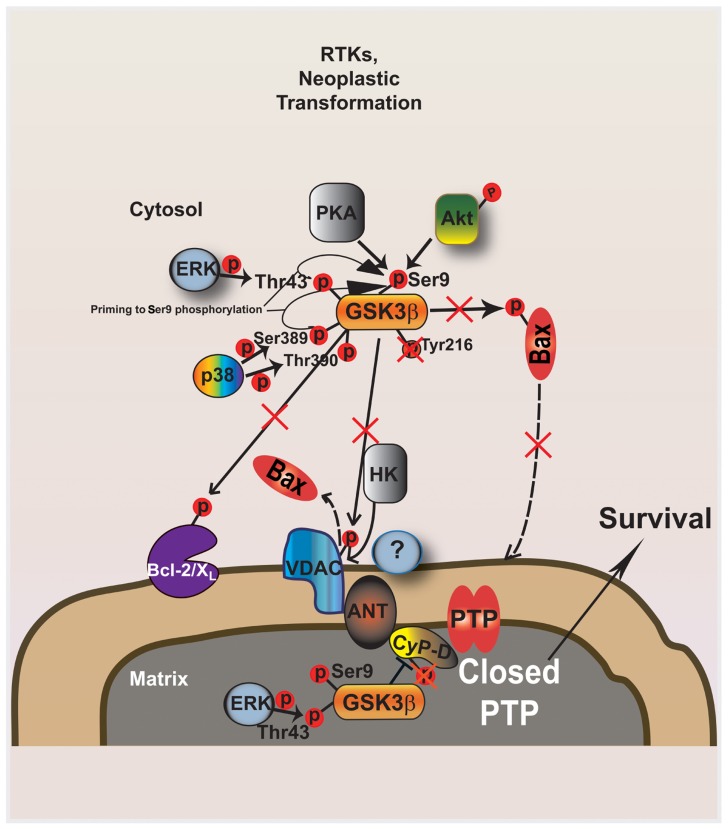
**GSK-3 modulates mitochondrial PTP opening downstream to kinase signaling activated during neoplastic transformation.** The active form of GSK-3 phosphorylates both Bax and VDAC: Bax is activated and migrates to the outer mitochondrial membrane (OMM), where it oligomerizes and induces membrane permeability; phosphorylated VDAC becomes a consensus site for Bax which displaces HK II from the same binding site. The mitochondrial fraction of GSK-3 can facilitate PTP opening by CyP-D phosphorylation. Ser phosphorylation of both cytoplasmic and mitochondrial GSK-3 by several kinases, such as AKT and ERK, critically contributes to defuse the mitochondrial apoptotic machinery downstream to ligand- or oncogenically activated receptor tyrosine kinases. GSK-3 inhibitory phosphorylation in enhanced by regulatory loops that involve ERK and p38 MAPK targeting other Ser and Thr residues on GSK-3. PTP inhibition is elicited both by dephosphorylated VDAC, which has high affinity for HK II and competitively displaces Bax from the OMM, and by active mitochondrial ERK, which inhibits mGSK-3 by Ser phosphorylation, in turn blocking CyP-D phosphorylation and PTP induction. Contextually, the absence of GSK-3-dependent Bax activation inhibits Bax relocation in the OMM.

GSK-3 activity is regulated in several ways: by inhibitory phosphorylation at a N-terminal Ser residue; by activating phosphorylation on a Tyr residue; following priming phosphorylation of substrates by other kinases; by subcellular localization and by binding to scaffold proteins in multimeric complexes (Jope et al., 2007). Ser phosphorylation within its N-terminal region (at Ser9 of GSK-3β or Ser21 of GSK-3α) is a major mechanism for inhibiting GSK-3**enzymatic activity (**Figure 1**), as it creates a “pseudosubstrate” which intramolecularly binds to a phosphoprotein binding pocket within the active site of the kinase, suppressing activity by occluding primed substrate access to the pocket (Woodgett and Cohen, 1984; Frame et al., 2001). Multiple transduction pathways that are constitutively activated in tumors converge on GSK-3 to induce phosphorylation at Ser9/21 residues. GSK-3 activity is inhibited by growth factor signaling either through the PI3K pathway (Stambolic and Woodgett, 1994; Shaw and Cohen, 1999) or the MAPK cascade (Saito et al., 1994; Brady et al., 1998); and in response to agonists that activate the protein kinase A (PKA) (Fang et al., 2000; Li et al., 2000) or the protein kinase C (PKC) (Ballou et al., 2001; Fang et al., 2002). All these signals contribute to the oncogenic process by removing the tonic inhibitory effect exerted by GSK-3 on a plethora of biochemical cascades. The phosphorylation state of these Ser residues is dynamic, as the effect of the kinases is counterbalanced by GSK-3 dephosphorylation mediated by protein phosphatase-1 (Zhang et al., 2003). This process is further modulated, as GSK-3 Ser9/21 phosphorylation is facilitated when the Thr43 and Ser389/Thr390 residues in GSK-3β are phosphorylated by ERK- and p38-MAPK, respectively. Conversely, GSK-3 enzymatic activity is maximal following phosphorylation on Tyr-216 or Tyr-279 of GSK-3β and GSK-3α, respectively. These Tyr residues are autophosphorylated (Lochhead et al., 2006), although the possibility that other tyrosine kinases are responsible for GSK-3 tyrosine phosphorylation cannot be ruled out (Cole et al., 2004).

Substrate specificity is influenced both by GSK-3 distribution in cell compartments (i.e., cytoplasm, nucleus, or mitochondria), and by its tendency to associate with other proteins in multimeric functional complexes. This is particularly important, as GSK-3 targets substrates that have already been primed, i.e., phosphorylated by another kinase present in the complex; the ideal phosphoacceptor site for GSK-3 is a Ser or Thr located four residues upstream to an already phosphorylated hydroxyamino acid (Woodgett and Cohen, 1984). An example is provided by the large molecular machinery that comprises Axin, adenopatous polypolis coli (APC), caseine kinase 1 (CK1), β-catenin, and GSK-3, which is engaged by the canonical Wnt signaling pathway and whose dysregulation is causative of diverse tumor types (Polakis, 2007). In this complex, co-localization of GSK-3 with a specific subset of binding proteins favors selective protein:protein interactions, leading to CK1-dependent priming of β-catenin, which is subsequently phosphorylated by GSK-3 and tagged for proteasomal degradation, thus blocking its transcription activity that favors cell proliferation (Wu and Pan, 2010).

## GSK-3 AND Bcl-2 FAMILY PROTEINS

GSK-3 contributes to the anti-apoptotic phenotype of cancer cells by controlling the mitochondrial localization and the activation status of a number of proteins of the cell death machinery, shaping the ability of cell death escape that hallmarks malignancies. Numerous proteins relevant to cell death [i.e., Mcl-1, Bcl-2, Bax, Noxa, voltage-dependent anion channel (VDAC), and adenine nucleotide transporter (ANT)] are board of GSK-3 and are located or can translocate in mitochondria. As a general rule, intrinsic apoptosis elicited by a variety of stress conditions that can be encountered by a malignant cell, such as withdrawal of growth factors, chemotherapeutics, or oxidant stress, is facilitated by active GSK-3 (Beurel and Jope, 2006). Thus, GSK-3 activity potentially counteracts neoplastic transformation. The phosphorylation of targets located on the external surface of mitochondria does not strictly require a mitochondrial localization of GSK-3, but it is plausible to envisage that the enzyme must be associated to outer membrane components. GSK-3 regulates several members of the B-cell lynphoma-2 (Bcl-2) protein family: it prompts phosphorylation-mediated proteasomal degradation of the anti-apoptotic protein myeloid cell leukemia-2 (Mcl-2) (Maurer et al., 2006; Ding et al., 2007a), whose expression correlates with Ser-phosphorylation dependent inactivation of GSK-3 in diverse cancer cell types (Ding et al., 2007b), whereas a decreased phosphorylation of potential GSK-3 target sequences on Bcl-2 itself contributes to its anti-apoptotic activation (Juhaszova et al., 2009). GSK-3 inhibition abolishes both the mitochondrial translocation and the conformational activation of the pro-apoptotic protein Bcl-2 associated X (Bax) through direct phosphorylation of a Ser residue on Bax found within a putative GSK-3 phosphorylation motif; and a constitutively active GSK-3 prompts Bax localization to mitochondria (Linseman et al., 2004; Ohori et al., 2008; Ge et al., 2012; Ngok-Ngam et al., 2013). In contrast with these observations, it was shown in human colorectal cancer cells that pharmacologic inhibition of GSK-3 elicited p53-dependent conformational activation of Bax, resulting in apoptosis induction (Tan et al., 2005). Moreover, treatment of melanoma cells with the multiple kinase inhibitor sorafenib activates GSK-3, leading to down-modulation of the pro-apoptotic Bcl-2 family member Noxa (Panka et al., 2008). These findings suggest that, at least in some neoplastic models, GSK-3 inhibition could enhance apoptosis.

## GSK-3 AND HEXOKINASE II

GSK-3 also regulates tumor cell survival by controlling mitochondrial binding of hexokinase, particularly hexokinase type II (HK II), which is highly expressed on the outer mitochondrial membrane (OMM) of most cancer cells. HK initiates the process of intracellular glucose utilization and it contributes to the Warburg effect, i.e., to the uncoupling between glycolysis enhancement and oxygen availability (Warburg, 1956; Hsu and Sabatini, 2008), supporting cell proliferation in the hypoxic conditions of primary tumor mass accrual. Association of HK II to the OMM is enhanced when GSK-3 is inactivated through phosphorylation by the survival kinase Akt, whose signaling is constitutively induced in most tumor types. Accordingly, activation of GSK-3 was shown to induce the release of HK II from the OMM (Mathupala et al., 2006; Robey and Hay, 2006). It was found that release of HK II from mitochondria induces apoptosis in hepatocellular carcinoma cells (Kim et al., 2007), and in glioma cells (Machida et al., 2006). We have observed both in tumor cell models and in cardiomyocytes that detachment of HK II from mitochondria by a selective peptide elicits opening of the inner membrane channel permeability transition pore (PTP), which irreversibly commits cells to death (Chiara et al., 2008). Thus, kinase cascades aberrantly boosted in neoplasms such as the PI-3K/Akt pathway funnel signals that inhibit GSK-3 and keep HK II bound to the mitochondrial surface, which maintains the PTP locked contributing to the anti-apoptotic phenotype of tumor cells; whereas GSK-3 activation lowers the threshold for cell death induction by favoring mitochondrial detachment of HK II and the ensuing PTP opening. The mechanism by which GSK-3 controls the mitochondrial localization of HK II remains unclear. It was proposed that GSK-3 phosphorylation of the VDAC on the OMM could induce cell death favoring the interaction between VDAC and the pro-apoptotic Bax/Bcl-2 homologous antagonist killer (Bak) proteins in conditions of nutrient starvation (Mathupala et al., 2006; Robey and Hay, 2006). However, the physiological role of GSK-3-mediated phosphorylation of VDAC is unknown, as mitochondrial displacement of HK II elicits cell death also in conditions of GSK-3 inhibition, in cells lacking any detectable binding between HK II and VDAC (Chiara et al., 2008), and in the absence of Bax and Bak (Majewski et al., 2004). Moreover, after Ser phosphorylation GSK-3 interacts with the mitochondrial ANT but not with VDAC (Nishihara et al., 2007), and this binding could play a role in protection from oxidative insults leading to PTP opening, at least in cardiomyocyte models.

## THE MITOCHONDRIAL POOL OF GSK-3

After the first detection of GSK-3 in mitochondria of rat cerebellum (Hoshi et al., 1995), a number of observations have clearly established that a fraction of the enzyme localizes in mitochondria. Mitochondrial GSK-3 (mGSK-3) contributes to the regulation of energy metabolism: it down-modulates both the Krebs cycle, by inactivating pyruvate dehydrogenase (Hoshi et al., 1996), and oxidative phosphorylation, by inhibiting NADH:ubiquinone oxidoreductase, i.e., respiratory chain (RC) complex I (King et al., 2008). As RC complexes are the main site of reactive oxygen species (ROS) production in the cell (Murphy, 2009), mGSK-3 is involved in the homeostatic redox equilibrium, whose dysregulation can lead to a number of pathological conditions. mGSK-3 inhibition activates an anti-oxidant response that reduces damage and promotes mitochondrial biogenesis during ischemic cerebral injury (Valerio et al., 2011), whereas it prevents ROS-dependent PTP opening in a model of hepatic ischemia/reperfusion (Varela et al., 2010). Tumour necrosis factor-alpha (TNF-α), whose signaling is constitutively enhanced during chronic liver inflammation, induces a ROS-dependent activation of mGSK-3 that causes depletion of mitochondrial DNA in human hepatic cells (Vadrot et al., 2012). mGSK-3 is the point of convergence of several transduction pathways that regulate PTP opening following ischemia/reperfusion in the heart (Juhaszova et al., 2004), including the survival kinases Akt and Erk1/2, PKCε, protein kinase G (PKG) and p70s6K (Hausenloy and Yellon, 2007); notably, effectiveness of ischemic pre- and post-conditioning in preserving cardiomyocyte viability requires mGSK-3 inhibition through Ser phosphorylation, which in turn inhibits the PTP in response to ROS or Ca^2^^+^ overload (Juhaszova et al., 2009; Miura and Miki, 2009; Miura et al., 2010). Accordingly, a significant increase in active, Ser-dephosphorylated mGSK-3 is observed during ischemia (Miura and Tanno, 2012).

The molecular mechanisms that regulate the mitochondrial pool of GSK-3 and in turn the PTP in cardiomyocytes could be relevant to neoplasms too, strongly contributing to the anti-apoptotic phenotype of tumor cells. Indeed, several data indicate that PTP dysregulation has a role in tumorigenesis, increasing resistance of neoplastic cells to a variety of stressful conditions such as exposure to chemotherapeutics, hypoxia, or detachment from the extracellular matrix (Rasola et al., 2010b). Even if the lack of a molecular characterization of the PTP hampers a thorough characterization of its modulation by mGSK-3, it is conceivable that mGSK-3 could contribute to PTP regulation both by acting as a downstream effector of diverse signaling pathways, and by changing mitochondrial ROS levels, as ROS are well-established PTP inducers (Rasola and Bernardi, 2011). Tumor cells are particularly exposed to the noxious effects of loss of redox homeostasis, as they are endowed with abnormally high ROS levels (Cairns et al., 2011). Thus, by modulating the PTP mGSK-3 could crucially affect the survival potential of neoplastic cells. Accordingly, it was observed that mGSK-3 activation enhances ROS production and apoptosis following treatment of neurons and of human neuroblastoma cells with complex I inhibitors (King et al., 2008; Petit-Paitel et al., 2009). Moreover, we and others have observed that cyclophilin D (CyP-D), a mitochondrial chaperone that regulates the PTP (Rasola and Bernardi, 2007), is directly phosphorylated by GSK-3 on Ser/Thr residues in tumor cell models (Rasola et al., 2010a; Traba et al., 2011) or in cells lacking mitochondrial DNA and characterized by a Warburg-like metabolic phenotype (Masgras et al., 2012). We found that a portion of ERK locates in the mitochondrial matrix, and mitochondrial ERK, which turned out to be constitutively active after v-Ki-Ras dependent transformation or in diverse neoplastic cell types, inhibits mGSK-3 by Ser phosphorylation, thus conferring resistance to death stimuli acting as PTP inducers (Rasola et al., 2010a). Moreover, ERK inhibition increased GSK-3-dependent phosphorylation of CyP-D and sensitization of PTP to opening, thus significantly abolishing tumor cell protection from apoptosis, whereas pharmacological inhibition of GSK-3 protected from PTP opening (Rasola et al., 2010a).

## GSK-3, MITOCHONDRIA AND CHEMOTHERAPY

GSK-3 displays a multiplicity of functions in distinct cellular compartments and in a variety of cell types, and it is involved in several disorders. This makes GSK-3 an interesting target for drug discovery, but at the same time considering GSK-3 as a therapeutic target exposes to the risk of undesired side effects, particularly when patients are treated in a chronic mode. During the last decade, a priority has been given to the search for GSK-3 inhibitors, and promising data exist for the treatment of neurological disorders and diabetes (Gould et al., 2006; Gao et al., 2011). In the field of cancer chemotherapy, GSK-3 mainly acts as a tumor suppressor by inhibiting many proto-oncogenic proteins and tumor development. Nonetheless, in neoplasms such as human ovarian, colon, hepatic and pancreatic carcinomas, some studies suggest that GSK-3 may actually exert a pro-neoplastic function, and inactivation of GSK-3 is associated with growth suppression of medullary thyroid cancer cells (Luo, 2009). As a consequence, targeting GSK-3 with drugs that could act as anti-neoplastic agents is an extremely complicated issue. For instance, GSK-3 displays a tumor suppressor activity in mammary tumors, and its activation causes sensitization to chemotherapeutics of breast cancer cells, but in colon cancer, where the enzyme is a tumor promoter, GSK-3 must be inhibited to increases the effect of chemotherapy (Luo, 2009). These paradoxical observations are probably explainable by the pleiotropy of GSK-3 functions, and a better comprehension of its molecular targets in the different subcellular locations could help to refine pharmacological approaches. The key role played by mitochondria in cell death regulation makes the mitochondrial function of GSK-3 a particularly attractive area of investigation. As an example, in hepatoma cells the effect of chemotherapy can be enhanced by pharmacological inhibition of the PI-3K signaling pathway, as this reactivates GSK-3 and facilitates mitochondrial translocation of Bax and the ensuing apoptosis (Beurel et al., 2005).

**FIGURE 2:**
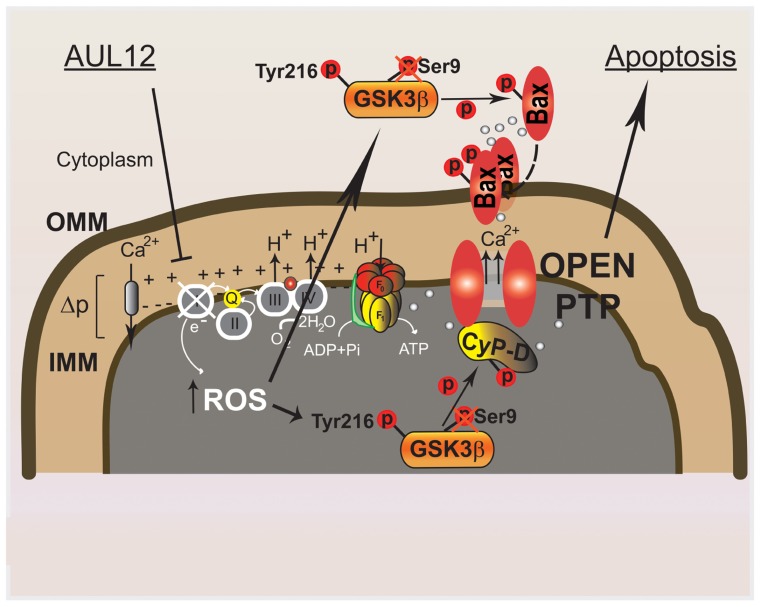
**Molecular mechanisms elicited by the recently synthesized gold-compound AUL12 to specifically induce cell death of tumor cells.** AUL12 inhibits the complex I of the mitochondrial respiratory chain thus eliciting an increase in ROS levels. The ROS surge reactivates the oncogenically inhibited GSK-3; mitochondrial GSK-3 phosphorylates CyP-D, unlocking the PTP blocked by oncogene signaling; cytosolic GSK-3 binds to and phosphorylates Bax, leading to its mitochondrial translocation. Mitochondrial Bax prompts mitochondrial permeabilization at least partially by inducing the PTP.

In this conceptual framework, we have recently characterized the mitochondrial effects of the Gold(III)-dithiocarbamato complex AUL12, a gold-based chemotherapeutic of new generation designed with the specific aim of improving selectivity, bioavailability, and efficacy of platinum-based compounds, diminishing their toxic side effects (Ronconi and Fregona, 2009). It was observed that AUL12 increases intracellular ROS levels (Saggioro et al., 2007); therefore, our idea was to target the increased ROS levels that characterize tumor cells. As cancer cells are forced to induce anti-oxidant defenses to set a novel homeostatic redox equilibrium, we reasoned that a further increase in ROS levels could overwhelm their residual anti-oxidant capabilities, triggering PTP opening and cell death in a selective way, i.e., without a major damage to non-transformed cells. We observed (Chiara et al., 2012) that AUL12 elicits a rapid burst of mitochondrial superoxide levels following inhibition of the RC complex I, which causes GSK-3 Tyr-phosphorylation and activation. The mitochondrial fraction of GSK-3 phosphorylates CyP-D, which in turn facilitates PTP opening, whereas the cytosolic GSK-3 interacts with Bax and prompts its mitochondrial translocation, where it contributes to PTP induction and tumor cell death (**Figure 2**). Notably, AUL12 was much less toxic on non-transformed cells and after *in vivo* administration (Marzano et al., 2011).

These findings provide evidence that targeting specific signaling pathways maintained by mitochondria in tumor cells allow to shut crucial mechanisms that shield neoplasms from the toxicity of many anti-neoplastic strategies, and pave the way for the design of a new family of chemotherapeutic compounds that sensitize cancer cells to chemotherapy.

## Conflict Of Interest Statement

The authors declare that the research was conducted in the absence of any commercial or financial relationships that could be construed as a potential conflict of interest.
